# Neighbourhood environment and dementia in older people from high-, middle- and low-income countries: results from two population-based cohort studies

**DOI:** 10.1186/s12889-020-09435-5

**Published:** 2020-09-01

**Authors:** Yu-Tzu Wu, Carol Brayne, Zhaorui Liu, Yueqin Huang, Ana Luisa Sosa, Daisy Acosta, Matthew Prina

**Affiliations:** 1 Department of Health Service and Population Research, Institute of Psychiatry, Psychology and Neuroscience, King’s College London, David Goldberg Centre, De Crespigny Park, London, SE5 8AF UK; 2 Department of Public Health and Primary Care, Cambridge Institute of Public Health, University of Cambridge, Cambridge, CB2 0SR UK; 3 Peking University Sixth Hospital, Peking University Institute of Mental Health, NHC Key Laboratory of Mental Health (Peking University), National Clinical Research Center for Mental Disorders (Peking University Sixth Hospital), Beijing, China; 4 National Institute of Neurology and Neurosurgery of Mexico, National Autonomous University of Mexico, Mexico City, Mexico; 5 Internal Medicine Department, Geriatric Section, Universidad Nacional Pedro Henriquez Ureña (UNPHU), Santo Domingo, Dominican Republic; 6 King’s Global Health Institute, King’s College London, London, SE5 8AF UK

**Keywords:** Dementia, Older people, Built environment, Local amenities, Green and blue spaces, Epidemiological cohorts, Geographic information system

## Abstract

**Background:**

A growing number of studies have explored how features of the neighbourhood environment can be related to cognitive health in later life. Yet few have focused on low- and middle-income countries and compared the results across different settings. The aim of this study is to investigate the cross-sectional associations between neighbourhood amenities and dementia in older people from high-, middle- and low-income countries.

**Methods:**

This study was based on two population-based cohort studies of people aged≥65: the Cognitive Function and Ageing Study II (CFAS II) in UK (*N* = 4955) and a subset of the 10/66 study in China, Dominican Republic and Mexico (*N* = 3386). In both cohorts, dementia was assessed using the Geriatric Mental State−Automated Geriatric Examination for Computer Assisted Taxonomy (GMS-AGECAT) algorithm. The 10/66 dementia diagnostic algorithm was also used as an additional criterion in the 10/66 study. Publicly accessible databases, Google Maps and Open Street Map, were used to obtain geographic information system data on distance to neighbourhood amenities, including lifestyle (cafés, libraries, movie theatres, parks), daily life (post offices, convenience stores), healthcare (hospitals, pharmacies) and percentages of local green and blue spaces within 400 and 800 m of participants’ residences. Multilevel logistic regression was used to investigate the associations between these environmental features and dementia adjusting for sociodemographic factors and self-rated health.

**Results:**

Living far from daily life amenities was associated with higher odds of dementia in both CFAS II (1.47; 95% CI: 0.96, 2.24) and the 10/66 study (1.53; 95% CI: 1.15, 2.04), while living far from lifestyle (1.50; 95% CI: 1.13, 1.99) and healthcare amenities (1.32; 95% CI: 0.93, 1.87) was associated with higher odds of dementia only in the 10/66 study. A high availability of local green and blue spaces was not associated with dementia in either cohort yet living far from public parks was associated with lower odds of dementia in CFAS II (0.64; 95% CI: 0.41, 1.00).

**Conclusions:**

The different relationships across cohorts may indicate a varying role for local amenities in diverse settings. Future research may investigate mechanisms related to these differences and social, cultural and historical influences on the interaction between neighbourhood amenities and older people.

## Background

Dementia has been recognised to be an important public health issue in ageing societies across the globe [1]. To reduce the risk of developing dementia in later life, a large body of studies has focused on modifiable lifestyle factors, such as physical activity and social engagement, or management of chronic conditions, such as hypertension and diabetes [2, 3]. Since lifestyle and health conditions can be influenced by the wider context of physical and social environment [4], a growing number of studies have explored how features of the neighbourhood environment are linked to cognitive health in later life and identified specific environmental features related to cognitive function and dementia using population-based cohorts of ageing populations [5, 6].

In recent years, the environmental impact on dementia, including air pollution, noise and features of the built environment, have been investigated in public health research [6, 7]. For the built environment, existing studies have mainly focused on two aspects: availability of green spaces [8–10] and local amenities such as food stores, libraries, community centres and recreational facilities [11–15]. Availability of these environmental features are thought to reduce stress and depressive symptoms and encourage physical activity and social interaction, creating a complex and stimulating environment that could increase cognitive reserve and reduce the risk of cognitive decline and dementia [16]. Not all studies have yielded consistent results. For example, cross-sectional results from the Multi-Ethnic Study of Atherosclerosis (MESA), a US-based cohort of 4716 older adults from six states, reported negative associations between cognitive function and the density of social and walking destinations such as postal offices, restaurants and beauty shops in local areas [13]. On the contrary, low availability of local food stores and restaurants was found to be associated with an increased risk of developing dementia over a three-year follow-up period in a population-based cohort of 49,511 older adults living in Japan [12]. These inconsistencies could be related to variation in research methods across studies, such as cross-sectional or longitudinal designs, sampling approaches, assessment methods for exposures, outcomes or other covariates and the availability of data for local amenities at different time points. While the impact of environmental features might be similar across settings, variation found in existing studies can be largely attributed to methodological differences. It is also possible that these diverse relationships reflect social and cultural influences on interaction with local amenities or other environmental features across different countries or settings. This can have strong implications on developing specific interventions to improve the local environment and support cognitive health in older people living in diverse settings. To disentangle the potential impacts of research methods from true social and cultural variation, it is necessary to use similar or comparable methods to investigate the associations in different study populations across countries or settings.

Although studies from high-income countries have reported complex relationships between environmental features and cognitive health in later life, there is a lack of evidence from low- and middle-income countries (LMICs). In recent years, many ageing cohorts have been established to collect in-depth data on cognitive function, health and risk factors in different older populations across the world [17] yet very few cohort studies have been designed to include environmental data in the investigations. At the same time, the rapid growth of computing technologies has facilitated the development of geographical information system (GIS) data, which inform characteristics of place [18]. Different types of GIS data have become accessible to the public, providing rich information on neighbourhood environments. Combining these two sources of data will enhance existing cohorts and create a cost-effective approach to advance research in the field.

Utilising online GIS data resources and existing ageing cohorts, the aim of this study is to investigate whether a higher availability of local amenities, green and blue spaces is associated with lower odds of dementia in older people living in the UK, China, Dominican Republic and Mexico. This study focused on whether similar relationships could be identified in these four high-, middle- and low-income countries when using comparable measures for environmental features, dementia and other sociodemographic factors.

## Methods

### Study population

This study was based on the Cognitive Function and Ageing Study II (CFAS II) [19] in the UK and a subset of the 10/66 study [20]. CFAS II is a population-based cohort of people aged 65 or above across three areas in England including Cambridgeshire, Nottingham and Newcastle-upon-Tyne. Participants in Cambridgeshire are considered to live in rural settings while the Nottingham and Newcastle-upon-Tyne sites mainly focused on urban areas. The baseline was conducted in 2008–2011 and a two-year follow-up was carried out between 2011 and 2013. The 10/66 study is a population-based cohort including 15,000 people aged 65 or over from China, India, Cuba, Dominican Republic, Venezuela, Mexico and Peru. The baseline interview was conducted in 2003–2007 and the follow-up waves were conducted between 2007 and 2010. The study areas were selected to identify urban districts with high density and deprivation in national or state capital cities and rural areas with a traditional lifestyle and low-density population. CFAS II was approved by the Cambridgeshire 4 Research Ethics Committee, National Health Service (NHS) Health Research Authority, and local research ethics committees (www.cfas.ac.uk/files/2015/07/Ethical-approvals-for-CFAS.pdf). The 10/66 study was approved by the King’s College London research ethics committee and in all local countries. All procedures comply with the ethical standards of the relevant national and institutional committees. Written informed consents were obtained from all participants. More detailed information on these two cohorts are provided elsewhere [19, 20].

Online mapping websites can only provide recent information about the environment and access to historical databases is not straightforward. The only exception is Google Maps in China, which has not been updated since 2010 due to legal restriction on geographic data in China. As the data on environmental features were collected in 2019, it was decided it would be more appropriate to use the latest investigations in these two cohorts. This study mainly focused on wave 2 investigations in CFAS II (2011–2013) and the follow-up phase of the 10/66 study (2007–2010) to examine the cross-sectional associations between environmental features and cognitive health in later life. Features of neighbourhood amenities were assumed to be stable between the time points of follow-up investigations and environmental data collection. This assumption could be plausible as some amenities such as public parks, libraries, hospitals, post offices, green and blue spaces were unlikely to change over time. In addition, a random sample of local amenities was selected from the UK cohort and 81.5% amenities could be found in Google Street View and Google Earth images which were close to the time point of the CFAS II follow-up wave (Supporting Information S1).

Due to limited funding, this study focused on all three CFAS centres in urban (Nottingham, Newcastle-upon-Tyne) and rural settings (Cambridgeshire) and three 10/66 sites including urban China, Dominican Republic and Mexico as these sites had more complete address information. Although China had urban and rural sites in Beijing, the rural site was excluded due to lack of complete residential addresses. Dominican Republic only had an urban site in Santo Domingo while Mexico included both urban (Mexico City) and rural sites (Morelos). The maps of study areas are provided in Figure S2, Supporting Information. The CFAS II wave 2 included 5288 interviewed participants. After excluding those who lived in institutions (*N* = 260) or who moved during the follow-up period (*N* = 264), 4955 UK participants from the urban (*N* = 3295) and rural areas (*N* = 1660) were left in the sample. For the 10/66 study, there were 3386 participants at wave 2 including 741 in urban China, 1190 in Dominican Republic, 748 in urban Mexico and 707 in rural Mexico.

### Assessment for dementia

Both CFAS II and the 10/66 study included the Geriatric Mental State (GMS) examination, which is a standardised interview for assessment of cognitive function and other neuropsychiatric syndromes in older people. The data from the GMS examination were entered into a diagnostic algorithm, the automated geriatric examination for computer assisted taxonomy (AGECAT), to generate a diagnosis indicating whether a person had dementia [21]. The GMS-AGECAT package provides an algorithmic approach to identify dementia cases in large epidemiological research and avoids being dependant on subjective clinical opinions that may lead to variation in diagnostic criteria and case identification across time or areas [19]. Despite the advantage of using algorithmic diagnosis, the substantial influence of education and culture on cognitive tests remained to be a concern for dementia assessment and diagnosis in LMIC settings [22]. To investigate the epidemiology of dementia in LMICs, the 10/66 Dementia Research Group developed the 10/66 dementia diagnostic algorithm for this multi-country cohort study [22]. In addition to the GMS examination, cognitive tests including the Community Screening Interview for Dementia and the modified 10-word list learning tasks from the Consortium to Establish a Registry for Alzheimer’s Disease battery were also carried out in the interviews and provided data for the 10/66 dementia diagnostic algorithm. This allowed the 10/66 study to generate dementia diagnoses based on the GMS-AGECAT algorithm and using the 10/66 dementia diagnostic algorithm. Although the 10/66 dementia diagnostic algorithm is considered to be more appropriate in LMIC settings than GMS-AGECAT, as widely reported in the 10/66 publications [20], the analysis here included both algorithms from the 10/66 study for an easier comparison with CFAS II.

### Individual factors

To reduce the potential impact of research methods heterogeneity, this study attempted to use the same measures in the two cohorts where possible. Individual sociodemographic factors including age, gender, education, social class/household assets and self-rated health were measured in the cohort interviews. Year of education in CFAS II was categorised into high (12 years or above), middle (10–11 years) and low (9 years or less) levels. In the 10/66 study, the category of education level was based on qualification: high (college or above), middle (secondary school), low (primary or none). Socioeconomic position was measured using social class in CFAS II and household assets in the 10/66 study. Social class in CFAS II was categorised based on the main occupation during participants’ lives and included six levels (I, II, III-NM, III-M, IV and V). The number of household assets (car, television, refrigerator, telephone, mains water, mains electricity and plumbed toilet) was used to indicate socioeconomic position in previous 10/66 work [23]. This measure was considered to be a more appropriate indicator for socioeconomic position in LMIC settings than occupation-based social class as most older participants were supported by their children or other family members. Self-rated health was measured using subjective rating of health status. In CFAS II, four option (excellent, good, fair and poor) were provided while there were five options (very good, good, moderate, bad and very bad) in the 10/66 study.

### Environmental factors

Google Maps and country-specific geocoding tools were used to convert UK postcodes and full address information in Chinese and Spanish into latitude and longitude coordinates. The geocoding results were verified with the sampling areas of cohort studies. Based on the coordinate information, eight types of local amenities for lifestyle (café, library, movie theatre and park), daily life (post office and convenience store) and healthcare (doctor/hospital and pharmacy) were obtained using Google Maps and its application program interface (Places API; https://cloud.google.com/maps-platform/places/). Amenities related to lifestyle included facilities which might support physical activity (park), social interactions (café) and cognitive reserve (library, movie theatre). Amenities for daily life included convenience stores, which can supply food, drinks and toiletries in local areas, and post offices, which provide postal or bank services. Amenities for healthcare services included doctors (GP surgeries in UK)/hospitals (in LMICs) and pharmacies. The distance to the nearest of each local amenity was calculated based on coordinates of participant’s residence and the specific amenity. The presence of different amenities within 400 m and 800 m were also generated. All data were managed using Stata 15.1.

Based on the land use layer from Open Street Map (www.openstreetmap.org), different types of green and blue spaces were identified and categorised into: recreational green space (park, allotment and recreational space), nature (forest and natural reserve) and blue space (river, lake or sea). Percentages of these three types of green and blue spaces within the 400 m and 800 m buffers were calculated using ArcGIS 10.6.1. The buffer sizes were chosen as they were considered to represent approximately 5- and 10-min walking distances [24]. More detailed information on generating environmental measures is provided in Supporting Information S1 and descriptive information is reported in Table S2.

Since the distributions of ‘distance to the nearest local amenities’ and ‘percentage of green and blue spaces’ were skewed, measures for environmental features were divided into tertiles based on the overall study population in each cohort. The tertiles indicated the shortest (T1) to longest (T3) distance to the nearest amenities and low (T1), middle (T2) and high (T3) availability of local green and blue spaces based on the percentages within 400 m and 800 m. The tertile ranges for local amenities, green and blue spaces are reported in Table S3.

### Analytical methods

Since the descriptive information showed that the two cohort studies had very different individual and environmental characteristics such as the percentage of dementia or distance to local amenities, the analysis here focused on whether similar patterns (i.e. relative changes in odds of dementia across levels of environmental features) could be identified in both CFAS II and the 10/66 study. The two cohorts were analysed separately. The associations between sociodemographic factors, self-rated health and dementia were examined using logistic regression models. Multilevel logistic regression modelling was used to investigate the associations between individual environmental features and dementia and a random intercept was included to take into account the nested-data structure (individuals living in the same households or buildings) [25]. Both unadjusted (Model 1) and adjusted associations accounting for sociodemographic factors and self-rated health listed in Table 1 (Model 2) are reported. Environmental features, which were found to be associated with dementia in Model 2, were included in one model to examine whether these amenities were independently associated with dementia (Model 3). All models included indicators of the three CFAS II centres or four 10/66 sites so any centre/site variation would be incorporated. The subgroup analyses further investigated whether the associations differed across centres/sites and individual socioeconomic levels. Interaction terms between local amenities, green and blue spaces and centre/site variables were fitted in the model. To examine whether individual socioeconomic positions modified the associations between environmental features and dementia, Model 3 further included interaction terms between local amenities and measures for socioeconomic levels (social class/number of assets), with stratified associations by socioeconomic levels being reported.

**Table 1:** Study populations in CFAS II and the 10/66 study

	CFAS II		10/66	
N		4955		3386
Dementia	GMS-AGECAT	189 (3.8)	GMS-AGECAT	705 (20.8)
	Missing	17	Missing	0
			The 10/66 algorithm	528 (15.6)
			Missing	9
Age	Mean (SD)	77.2 (6.7)	Mean (SD)	77.8 (6.4)
Gender	Men	2357 (47.6)	Men	1177 (34.8)
	Women	2598 (52.4)	Women	2207 (65.2)
	Missing	0	Missing	2
Education	High: 12 years or above	1207 (24.6)	High: college or above	605 (17.9)
	Middle: 10–11 years	2615 (53.2)	Middle: secondary	678 (20.1)
	Low: 9 years or less	1095 (22.3)	Low: primary or none	2093 (62.0)
	Missing	38	Missing	10
Social class	I/II	1414 (29.5)		
	III-NM	1339 (27.9)		
	III-M	1251 (26.1)		
	IV/V	788 (16.4)		
	Missing	163		
Number of household assets			0–3	1873 (55.3)
			4–5	1092 (32.3)
			6–7	421 (12.4)
Self-rated health	Excellent	1065 (21.7)	Very good	469 (13.9)
	Good	2618 (53.3)	Good	980 (29.0)
	Fair	1017 (20.7)	Moderate	1730 (51.1)
	Poor	216 (4.4)	Bad	169 (5.0)
			Very bad	35 (1.0)
	Missing	39	Missing	3
Urban/rural settings	Urban	3295 (66.5)	Urban	2679 (79.1)
	Rural	1660 (33.5)	Rural	707 (20.9)

*GMS-AGECAT* Dementia assessed based on the Geriatric Mental State−Automated Geriatric Examination for Computer Assisted Taxonomy algorithm; The 10/66 algorithm: dementia assessed based on the 10/66 dementia diagnostic algorithm

Multiple testing was an important concern. Although Bonferroni correction may be used to address multiple testing, this approach has been suggested to further reduce statistical power and increase Type II error [26]. To avoid identifying false positive results based on *p*-values, the judgement was based on the direction of associations (potential increasing or decreasing patterns), effect sizes and 95% confidence intervals. Since the percentage of missing data was low (< 10%) in the two cohorts, the results of complete case analysis (*N* = 4739 for the CFAS II; *N* = 3374 for the 10/66 study) are reported. Sensitivity analyses using multiple imputation were also carried out to test the impact of missing data on the results. All variables included in the modelling were used to generate 10 imputed datasets. Logistic regression modelling without a random intercept was applied to Model 3 and estimates from the imputed datasets were combined using Rubin’s rule.

## Results

Table 1 shows descriptive information on the study populations by CFAS II and the 10/66 study. The mean age was 77.2 years in CFAS II and 77.8 years in the 10/66 study. Both cohorts had a higher proportion of women than men. Most participants in the 10/66 study had none or primary education (62.0%). In CFAS II, over half of participants completed secondary education (10–11 years). In the 10/66 study, over 50% of participants only had up to three assets in their households.

Based on the GMS-AGECAT algorithm, the percentage of people with dementia was 3.8% (*N* = 189) in CFAS II and 20.8% (*N* = 705) in the 10/66 study. Based on the 10/66 dementia diagnostic algorithm, 528 (15.6%) people were identified to have dementia and the overlap of two diagnoses was 399 (11.8%). Among individual factors, older age, lower education and socioeconomic levels and poorer self-rated health were associated with higher odds of dementia (Table S4). The intraclass correlation was estimated to be 0.15 in CFAS II and < 0.01 in the 10/66 study when adjusting for all individual level factors.

**Fig. 1:**
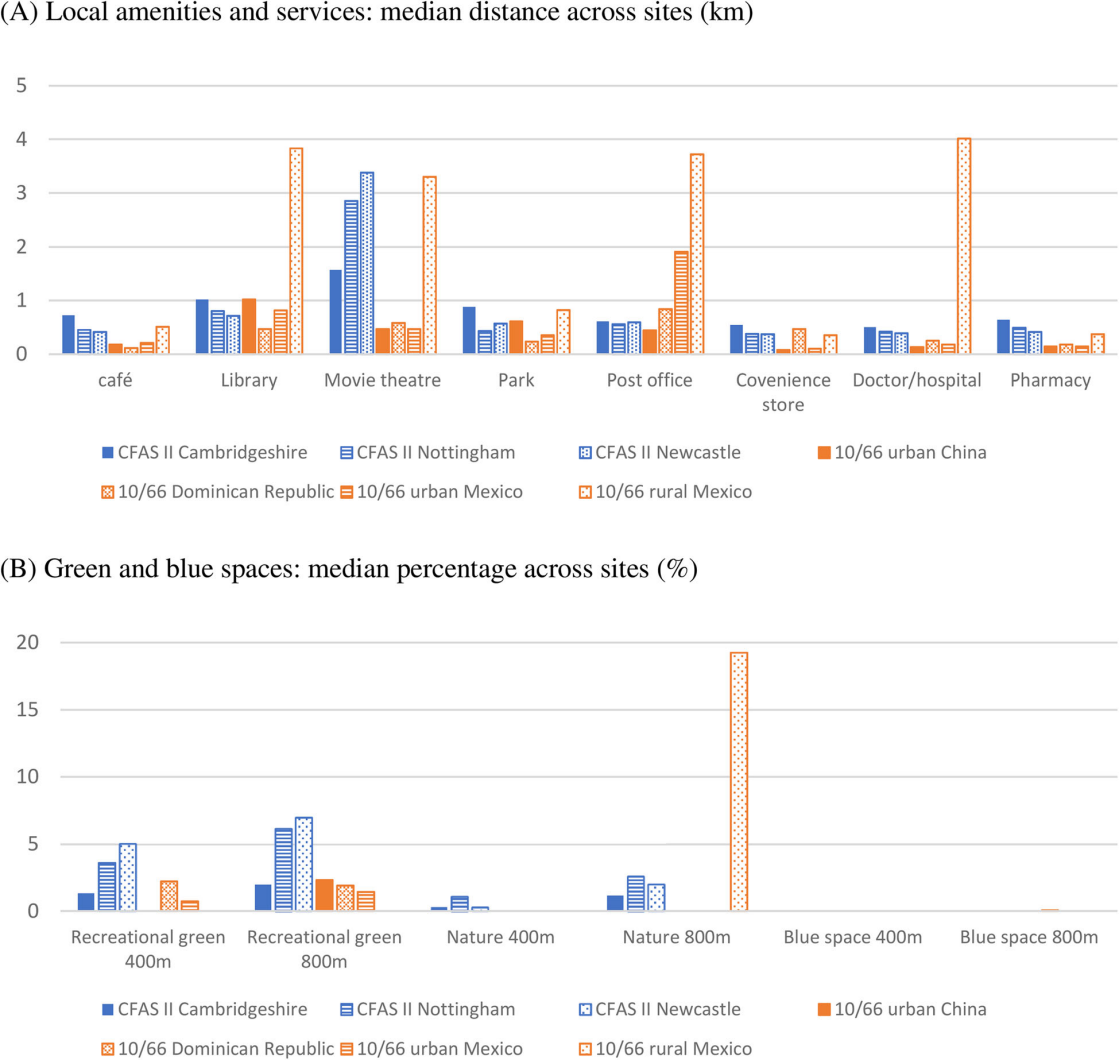
Distributions of distance to local amenities and percentage of green/blue spaces across study sites

Figure 1a shows the median distance to eight amenities in the three CFAS II centres and the four 10/66 sites. In CFAS II, the median distance to the nearest amenities was less than 1 km apart from movie theatre. The 10/66 rural Mexico site had generally longer distance to local amenities compared to other sites. Figure 1b reports the median percentage of local green and blue spaces within 400 m and 800 m across sites. The median percentages of recreational green spaces were higher in CFAS II (3.1% for 400 m; 4.5% for 800 m) than in the 10/66 study (0.7% for 400 m; 1.5% for 800 m). The percentages of nature green spaces were particularly high in rural Mexico (19.2%). The percentages of blue spaces were generally low or did not exist in the local areas of the participants.

Table 2 shows the associations between distance to the nearest local amenities and dementia in CFAS II and the 10/66 study and the associations for the presence of local amenities and green/blue spaces are reported in Table S5. In CFAS II, the associations were generally weak apart from park and post office (Table 2). Living far from public parks was associated with lower odds of dementia (0.64; 95% CI: 0.42, 0.95) and the effect sizes were not attenuated after adjusting for sociodemographic factors and self-rated health. Living far from a post office was associated with higher odds of dementia and the adjusted odds ratio was 1.47 (95% CI: 0.96, 2.24). For healthcare amenities, living in areas with a longer distance to a GP surgery or pharmacy was not associated with dementia after adjustment for individual factors. No associations were found for local green/blue spaces.

**Table 2:** The associations between local amenities, green and blue spaces and dementia (based on the GMS-AGECAT algorithm or the 10/66 dementia diagnostic algorithm) in CFAS II and the 10/66 study

		CFAS II (GMS-AGECAT)		10/66 study (GMS-AGECAT)		10/66 study (the 10/66 algorithm)	
		Model 1	Model 2	Model 1	Model 2	Model 1	Model 2
		OR (95% CI)	OR (95% CI)	OR (95% CI)	OR (95% CI)	OR (95% CI)	OR (95% CI)
**Local amenities**							
*Lifestyle*							
Café	T1	–	–	–	–	–	–
	T2	1.06 (0.73, 1.53)	1.03 (0.69, 1.54)	1.00 (0.80, 1.26)	1.04 (0.82, 1.33)	0.89 (0.70, 1.13)	0.88 (0.68, 1.15)
	T3	1.00 (0.68, 1.48)	1.07 (0.69, 1.64)	1.69 (1.30, 2.19)	1.50 (1.13, 1.99)	1.49 (1.12, 1.99)	1.34 (0.93, 1.83)
Library	T1	–	–	–	–	–	–
	T2	1.03 (0.71, 1.50)	1.01 (0.68, 1.52)	1.32 (1.06, 1.66)	1.25 (0.99, 1.58)	1.23 (0.96, 1.57)	1.16 (0.90, 1.50)
	T3	1.01 (0.69, 1.47)	1.15 (0.76, 1.74)	1.63 (1.23, 2.17)	1.28 (0.95, 1.73)	1.21 (0.89, 1.64)	1.00 (0.73, 1.39)
Movie theatre	T1	–	–	–	–	–	–
	T2	0.99 (0.68, 1.47)	0.97 (0.64, 1.48)	1.09 (0.86, 1.37)	1.03 (0.82, 1.31)	1.22 (0.96, 1.55)	1.18 (0.92, 1.51)
	T3	1.21 (0.81, 1.79)	1.19 (0.77, 1.84)	1.20 (0.93, 1.55)	1.09 (0.84, 1.42)	1.09 (0.82, 1.44)	1.00 (0.74, 1.36)
Park	T1	–	–	–	–	–	–
	T2	0.87 (0.61, 1.24)	0.89 (0.61, 1.32)	0.86 (0.69, 1.08)	0.87 (0.69, 1.09)	0.78 (0.61, 1.00)	0.77 (0.59, 0.99)
	T3	0.64 (0.42, 0.95)	0.64 (0.41, 1.00)	0.99 (0.75, 1.30)	0.88 (0.66, 1.17)	1.14 (0.84, 1.55)	0.99 (0.72, 1.36)
*Daily life*							
Post office	T1	–	–	–	–	–	–
	T2	1.23 (0.84, 1.80)	1.31 (0.86, 1.99)	1.15 (0.87, 1.51)	1.14 (0.84, 1.54)	0.77 (0.57, 1.03)	0.78 (0.57, 1.07)
	T3	1.20 (0.82, 1.76)	1.47 (0.96, 2.24)	1.79 (1.38, 2.31)	1.53 (1.15, 2.04)	1.30 (0.96, 1.75)	1.23 (0.88, 1.72)
Convenience store	T1	–	–	–	–	–	–
	T2	1.00 (0.69, 1.45)	1.03 (0.69, 1.54)	0.93 (0.73, 1.18)	0.93 (0.72, 1.20)	1.19 (0.90, 1.56)	1.18 (0.89, 1.58)
	T3	0.99 (0.67, 1.44)	1.04 (0.69, 1.59)	1.20 (0.90, 1.61)	1.11 (0.82, 1.50)	1.46 (1.05, 2.03)	1.46 (1.03, 2.07)
*Healthcare*							
Doctor/hospital	T1	–	–	–	–	–	–
	T2	1.02 (0.71, 1.46)	0.96 (0.64, 1.42)	0.95 (0.75, 1.21)	0.92 (0.72, 1.17)	1.14 (0.89, 1.47)	1.08 (0.83, 1.40)
	T3	0.81 (0.55, 1.19)	0.92 (0.60, 1.39)	1.05 (0.77, 1.43)	1.06 (0.78, 1.46)	1.32 (0.94, 1.85)	1.32 (0.93, 1.87)
Pharmacy	T1	–	–	–	–	–	–
	T2	1.09 (0.76, 1.54)	1.12 (0.76, 1.65)	0.97 (0.77, 1.22)	0.98 (0.78, 1.24)	1.17 (0.92, 1.49)	1.15 (0.89, 1.48)
	T3	0.69 (0.46, 1.04)	0.76 (0.49, 1.19)	1.07 (0.84, 1.35)	1.01 (0.80, 1.29)	1.32 (1.02, 1.71)	1.25 (0.95, 1.64)
**Green/blue spaces**							
*400 m*							
Recreational green	L	–	–	–	–	–	–
	M	1.00 (0.67, 1.48)	0.93 (0.62, 1.39)	0.92 (0.71, 1.18)	1.07 (0.83, 1.38)	0.76 (0.57, 1.00)	0.87 (0.65, 1.18)
	H	1.36 (0.93, 1.99)	1.24 (0.84, 1.84)	0.66 (0.49, 0.88)	0.89 (0.66, 1.20)	0.65 (0.48, 0.87)	0.82 (0.60, 1.15)
Nature	L	–	–	–	–	–	–
	M	0.99 (0.68, 1.44)	1.04 (0.70, 1.53)	0.98 (0.74, 1.30)	0.97 (0.73, 1.28)	1.00 (0.71, 1.41)	1.00 (0.71, 1.41)
	H	0.92 (0.64, 1.33)	0.99 (0.68, 1.46)				
Blue space	None	–	–	–	–	–	–
	Any	1.02 (0.68, 1.52)	1.07 (0.70, 1.62)	1.07 (0.72, 1.61)	1.09 (0.73, 1.64)	0.86 (0.55, 1.33)	0.86 (0.55, 1.36)
*800 m*							
Recreational green	L	–	–	–	–	–	–
	M	1.30 (0.86, 1.95)	1.21 (0.79, 1.85)	1.07 (0.78, 1.47)	1.10 (0.79, 1.53)	0.98 (0.91, 1.62)	0.99 (0.70, 1.42)
	H	1.34 (0.84, 2.12)	1.24 (0.77, 2.00)	0.69 (0.49, 0.95)	0.82 (0.58, 1.15)	0.80 (0.57, 1.11)	0.92 (0.65, 1.31)
Nature	L	–	–	–	–	–	–
	M	1.01 (0.70, 1.45)	1.03 (0.70, 1.50)	0.99 (0.74, 1.33)	0.90 (0.67, 1.22)	1.09 (0.76, 1.58)	0.99 (0.67, 1.45)
	H	0.86 (0.59, 1.27)	0.92 (0.61, 1.37)				
Blue space	None	–	–	–	–	–	–
	Any	1.04 (0.76, 1.41)	1.05 (0.76, 1.45)	1.10 (0.82, 1.46)	1.12 (0.85, 1.49)	1.05 (0.77, 1.44)	1.12 (0.81, 1.53)

All models adjusted for centre (CFAS II) or site (10/66); Model 1: unadjusted; Model 2: adjusted for age, gender, education, social class/assets and self-rated health; T1, T2, T3: first, second and third tertile of distance to the nearest local amenities; H, M, L: high, middle and low percentages of green and blue spaces within 400 m or 800 m by tertiles

In the 10/66 study, higher odds of dementia based on the GMS-AGECAT algorithm was associated with longer distance to café, library, movie theatre and post office but the associations for library and movie theatre were attenuated when adjusting for sociodemographic factors and self-rated health (Table 2). The adjusted odds ratio was 1.50 (95% CI: 1.13, 1.99) for café and 1.53 (95% CI: 1.15, 2.04) for post office. When using the 10/66 dementia diagnostic algorithm, higher odds of dementia was associated with longer distances to cafés (1.34; 95% CI: 0.93, 1.83), convenience stores (1.46; 95% CI: 1.03, 2.07) and doctors (1.32; 95% CI: 0.93, 1.87) after adjustment for individual factors. The percentage of local green/blue spaces was not associated with dementia.

**Fig. 2:**
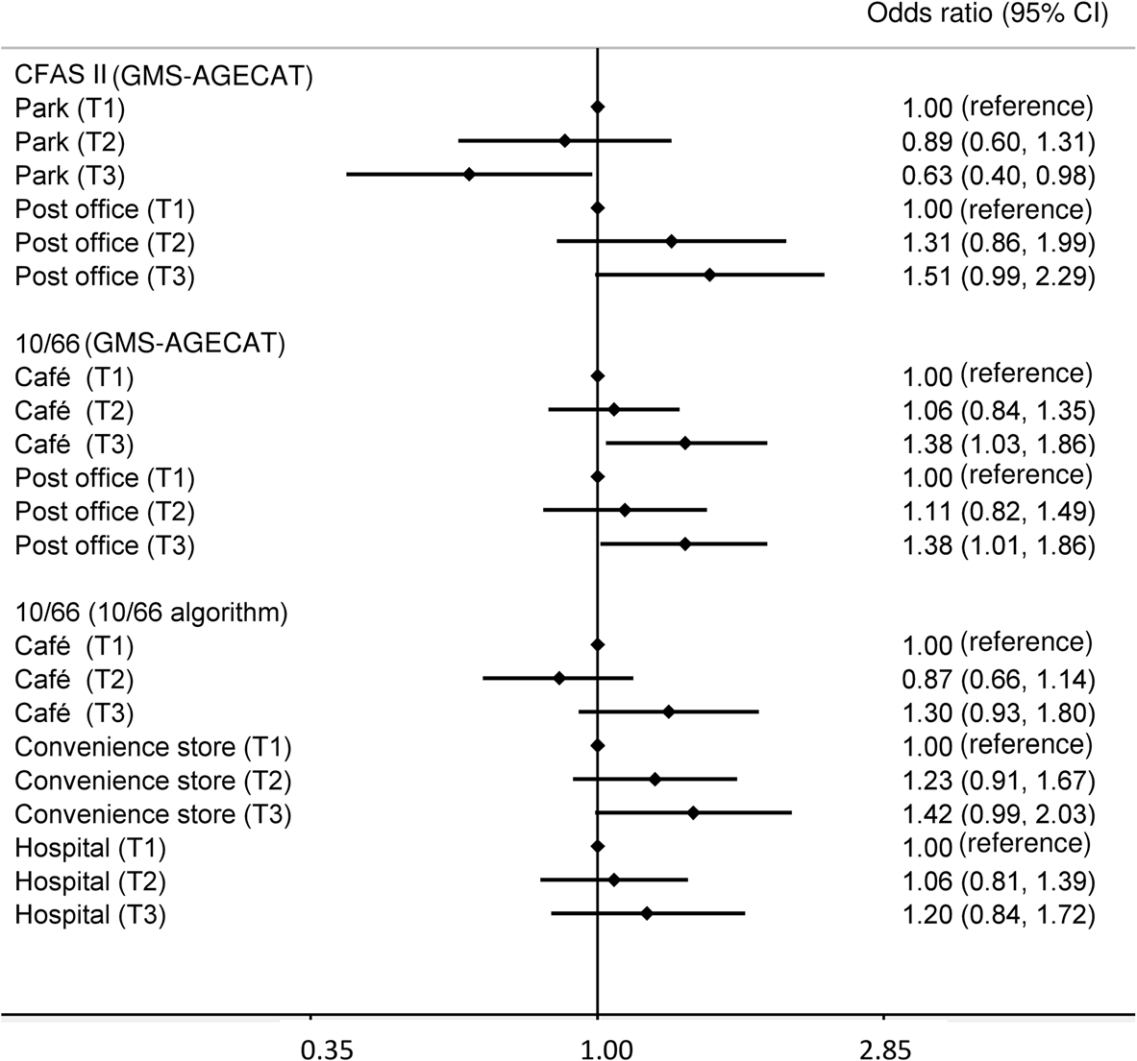
Results of the full models (Model 3) including multiple local amenities (adjusted for age, gender, education, social class/assets, self-rated health and centre/site; T1, T2, T3: first, second and third tertile of distance to the nearest local amenities)

Figure 2 reports the results of full models incorporating all local amenities which were found to be associated with dementia and adjusting for individual factors. Most amenities showed similar effect sizes in the models for CFAS II and the 10/66 study when using the GMS-AGECAT algorithmic diagnoses. In the model for the 10/66 dementia diagnostic algorithm, the effect size for proximity of hospital slightly reduced when taking the data on café and convenience store into account.

Table S6 reports the relationships between local amenities, green/blue spaces and dementia based on the GMS-AGECAT algorithm by the three CFAS II centres and the four 10/66 sites and Table S7 shows the stratified associations by individual socioeconomic levels. Although some effect sizes varied across these subgroup analyses, confidence intervals were generally wide and overlapped due to small sample sizes. Table S8 reports the imputed results of logistic regression modelling. The estimates were similar to the main analysis reported in Fig. 2.

## Discussion

### Main findings

Drawing on online GIS resources, this study provided evidence on the associations between local amenities, green and blue spaces and dementia in older people living in diverse settings. Using similar measurements and analytical methods across the UK and LMIC cohorts, some similar and varying trends were observed. In both cohorts, living far from daily life amenities (post office or convenience store) was associated with higher odds of dementia but the associations for lifestyle and healthcare amenities were only found in older people living in LMIC settings. A higher availability of local green and blue spaces was not found to be associated with dementia in either cohorts.

### Strengths and limitations

This study was based on existing data from two population-based cohorts in the UK and three LMICs and included older people living in diverse settings. These two studies had very different response rates, characteristics of study populations, geographical settings and prevalence of dementia. To reduce the potential impact of research methods heterogeneity on the results, the same approach was used to collect data on environmental features across cohorts and similar measures for dementia and sociodemographic factors were identified in the two studies. Instead of using census units or administrative districts in specific countries, the approach presented here was able to identify environmental features close to participants’ residences.

Due to the availability of data on environmental features, this study only focused on cross-sectional associations using the latest waves of investigations. The populations studied were based on those who survived and remained in the follow-up waves and are unlikely to be representative of the general older population unless extensive weighting back to the original sampling is undertaken. Although the results cannot imply causal directions, similar measurement and analytical methods used in this study allowed possible comparison of cross-sectional relationships across cohorts. The assumption that environmental features remained similar between time points of follow-up investigations and environmental data collection might be uncertain. Some LMICs might had dramatic environmental changes due to large scale infrastructure projects, urban planning or development. This could have attenuated any true associations as the latest environmental features would be likely to have had limited impact on cognitive health. Despite the same methods used to measure environmental features, some amenities such as public parks and hospitals might be more stable over time than other amenities. Variation in measurement errors might affect the identification of possible associations. This study used 400 m and 800 m buffers as a proximate activity space for older people in their daily life. Although most existing studies also generated buffers based on participants’ residences [11, 12], whether older people visited these green and blue spaces or travelled outside of local areas is not known. Although physical distances to the nearest amenities were measured in this study, other features of built environment such as availability of public transport, pavement conditions and street network might also affect the accessibility to amenities including those that might be at a distance [27, 28]. In addition, social factors such as coverage of healthcare insurance, crime and social capitals, might also influence the use of local amenities [29, 30]. Air pollution is closely linked to features of the built environment and might also affect the risk of developing dementia [7]. However, these environmental measures were not available in this study. Behavioural factors such as physical activity and social network could also be potential mediators on the pathway between environmental features and dementia [6, 16]. Yet it was difficult to identify comparable measures across the two cohorts. Dementia diagnosis was made using algorithmic approaches and did not consider longitudinal changes in cognitive function over time. With only small numbers of people meeting the criteria of algorithmic diagnoses, limited statistical power led to wide confidence intervals in the modelling. In particular, providing robust estimates for subgroup analyses on centre/site or socioeconomic levels was not possible. Given the large differences between the two cohorts, this study categorised environmental measures into tertiles and focused on relative changes in odds of dementia across levels of environmental features in the two cohorts. However, categorisation of environmental measures might affect identification of heterogeneity within categories and limit direct comparison with other studies.

### Interpretation of findings

The findings of this study echo the complexity of relationships reported from earlier studies. Research from US, Japan and Hong Kong has included different measures related to local amenities, with inconclusive results for cognitive health in later life [11–13]. Here, living in an area with poor access to daily life amenities (post offices or convenience stores) was found to be associated with higher odds of dementia in both cohorts. This type of amenities might support older people to manage their daily life such as paying bills and food shopping and could increase physical, social and cognitive activity [4, 31]. However, a US-based study found a negative association between density of walking destinations (postal services, eating or dining places) and cognitive function in older adults aged 45 or above [13]. In both UK and LMIC cohorts, distance to library was not associated with dementia after adjusting for individual level factors. In contrast a recent study based on 21,008 participants aged 65 or above in Hong Kong reported that accessibility to libraries, which incorporated measures for distance and collection size, was associated with lower odds of dementia [11]. Inconsistent associations between local amenities and dementia were also observed in the two cohorts from UK and LMICs. Several amenities for lifestyle, daily life and healthcare services were found to be associated with dementia in the LMIC cohort but not in the UK cohort. In addition to variation in methodologies and characteristics of study populations, the differences across studies and cohorts might be related to environmental, cultural and social factors in wider contexts. Other infrastructures and facilities such as public transport or pavement conditions might reduce the impact of physical distance and make distant amenities and services more accessible [27, 28]. These infrastructures are generally more comprehensive in high-income countries than LMICs. There will be historical, economic, cultural and social influences in the configuration of services in relation to population [32] and this is likely to modify the relationship between local amenities and older residents in different settings and across time.

Although other population-based cohorts from the UK have reported that a high availability of local green spaces was related with a slow rate of cognitive decline in later life [8, 9], the results from this study did not support benefits of green space on cognitive health in later life. Instead, lower odds of dementia were found to be related to longer distances to public parks in CFAS II. One possible explanation for this reverse relationship could be that living close to parks might reflect difficult access to other amenities. Although the effect sizes of public parks in the full model including multiple local amenities remained similar (Fig. 2), large areas of green space near residences might cause difficulties for travelling, or be associated with poor public transport infrastructures leading to isolation. Another possible explanation might be related to the variation in measurement errors across different amenities [33]. Compared to other local amenities, public parks are more likely to have been measured accurately, also remaining more consistent over time. Greater measurement errors in other amenities might dilute the strength of associations with dementia.

### Future research direction

The diverse relationships across cohorts may indicate varying roles for local amenities in different settings and have complicated implications on supporting cognitive health in later life. To develop potential interventions and create a supportive environment for the fast-growing older populations across the world, factors related to this variation need to be better understood, including how older people use amenities and services in local areas. Qualitative or mixed methods research designs would provide insights into older people’s experiences [28] and novel technologies such as global positioning system and mobile electroencephalogram equipment could be utilised in quantitative research and provide dynamic data on mobility, activity and real-time responses to environments [34, 35]. Cross-country studies using the same research methods are important to clarify how cultural, social and other environmental factors might modify the relationships between the built environment and the health of older people [36].

Integrating online GIS resources into existing ageing cohorts can be a promising approach, which adds values to both geographical and epidemiological datasets [37]. Although publicly accessible databases can provide a wide range of environmental data across the world, these data might be collected at different time points and could not match cohort investigations in the past. Since features of the neighbourhood environment can change over time due to economic development, urbanisation or deindustrialisation in many societies, obtaining historical data on environment can be crucial to facilitate longitudinal analysis and strengthen causal inferences. This will also create opportunities to investigate the potential impact of environmental changes and the cumulative effect of environmental exposures throughout different life stages [38], advance research evidence to inform population-level interventions that support cognitive health across the lifecourse.

## Conclusions

Availability of local amenities was associated with dementia but the relationships varied across older populations in the UK and LMICs. The different relationships across cohorts may indicate a varying role of local amenities in diverse settings. Future research may investigate mechanisms related to these differences and develop possible interventions to support cognitive health in later life.

## Supplementary information

**Additional file 1.**

## Data Availability

The datasets used during the current study are available from the corresponding author on reasonable request.

## Abbreviations

AGECAT: Automated geriatric examination for computer assisted taxonomy
CFAS II: Cognitive Function and Ageing Study II
GIS: Geographic Information System
GMS: Geriatric Mental State examination
GP: General Practitioner
LMICs: Low- and middle-income countries
