# Supplementary material for: Neighbourhood environment and dementia in older people from high-, middle- and low-income countries: results from two population-based cohort studies
Source: BMC Public Health. 2020 Sep 1;20:1330. doi: 10.1186/s12889-020-09435-5 (PMC7465327; doi:10.1186/s12889-020-09435-5)
Supplement: Supplementary file 1 — Additional file 1. [file 12889_2020_9435_MOESM1_ESM.docx]

**Neighbourhood environment and dementia in older people from high-, middle- and low-income countries: results from two population-based cohort studies**

**Supporting Information**

S1. Environmental measures

S2. Additional results

**S1. Environmental measures**

Figure S1 shows the process of integrating data on local amenities and services into the Cognitive Function and Ageing Study II (CFAS II) and the 10/66 study. Postcode or address information from the two cohort participants were first converted into latitude and longitude coordinates through Google Maps or country-specific converters. Based on the coordinate information, data on local amenities were extracted using Google Place API searches and different layers of geographic information system (GIS) data were obtained from Open Street Map. Distances between participants’ residences and local amenities were calculated to identify distance to the nearest amenities and the presence of local amenities within 400m and 800m. Percentages of green/blue spaces with 400m and 800m were estimated using the ArcGIS software. More detailed information is provided in the following sections.

**Figure S1. The process of integrating data on local services and green/blue spaces into the Cognitive Function and Ageing Study II (CFAS II) and the 10/66 study**

Cohort data - address information

CFAS II: UK postcode

The 10/66 study: Address text

Google Maps, country-specific converter

Latitude/longitude coordinates

Google Places API + Stata programme

Open Street Map + ArcGIS

Eight types of local services and resources (the nearest 20 results for each coordinate)

Stata programme

Calculate distances between participants’ residences and the nearest services or amenities

GIS data on land use: park, allotment, recreation space, forest, natural reserve and waterways

Calculate percentages of green and blue spaces within 400m and 800m buffers of participants’ residences

ArcGIS

**Geocoding**

In the UK, postcode information can be transferred into latitude and longitude using the online converter (<https://gridreferencefinder.com/postcodeBatchConverter/>). Postcode information from the CFAS II participants were converted to latitude and longitude based on the World Geodetic System 84 (WGS-84). Since postcode information might not be available in low- and middle-income countries, text of residential address in the 10/66 study was used to identify coordinate information. The free text of addresses was used to extract information on house number, street, district (if applicable) and city/village. For the 10/66 China site, the Chinese addresses were transferred into coordinates using a country-specific converter (<https://maplocation.sjfkai.com/>) and a special coordinate system for China, GCJ-02 (colloquially Mars Coordinates), was obtained for all urban participants. The GCJ-02 system was also used in Google Maps for China. Since participants in the rural China site did not have complete addresses and some villages were moved due to airport construction, the coordinate information was not applicable for these participants. For the 10/66 Dominican Republic and Mexico sites, the Spanish addresses were converted into latitude and longitude (WGS-84) using Google Maps. The geocoding results were verified with the study catchment areas.

**Local amenities**

The Google Places application program interface (API) is a tool which can be used to identify specific types of amenities within given areas or locations and obtain detailed information name, address and coordinates from the Google Maps database (<https://cloud.google.com/maps-platform/places/>). The maximum search radius is 50000 metre and each search reports up to 20 records and the priority can be determined by distance or prominence (based on Google’s index).

Due to limited funding, the searches were carried out to identify eight types of local amenities for lifestyle (café, library, movie theatre and park), daily life (post office and convenience store) and health care (doctor/hospital and pharmacy). Amenities related to lifestyle included facilities which might support physical activity (park), social interactions (café) and cognitive reserve (library, movie theatre). Amenities for daily life included convenience store, which can supply food, drinks and toiletries in local areas, and post office, which provides postal or bank services. Amenities for health care included doctor/hospital and pharmacy. Although GP surgery and clinics in UK are categorised under ‘doctor’ in Google Maps, these LMICs might not have primary care system and therefore ‘hospital’ would be more appropriate for general health care. In these LMICs, the ‘doctor’ category in Google Maps was more likely to identify private health services, such as cosmetic surgery and weight management centres,

For each amenity, the nearest 20 records to participants’ residence were obtained from Nearby searches in Google Places API. Name, latitude and longitude information for identified amenities was formatted into tables via Stata programmes (modified version of *goolgeplaces.ado*). Straight distances between each participant’s residence and the nearest amenities were calculated based on coordinate information. The distances were used to identify the numbers and presence of local amenities within 400m and 800m. All data were managed using Stata 15.1.

**Green and blue spaces**

Open Street Map (OMS, [www.openstreemap.org](http://www.openstreemap.org)) is a website providing open-accessed maps across the world. The online maps can be transferred into GIS data, which contain point, line and polygon layers for spatial analysis. The GIS data files (.shp, .osm.pbf, .osm.bz2) for specific countries or regions can be downloaded from the GEOFARBRIK website (<http://download.geofabrik.de/>). The GIS data for the CFAS II (Cambridgeshire, Nottingham and Newcastle upon Tyne) and the 10/66 study areas (Beijing, Santo Domingo, Mexico City and Morelos areas) were downloaded in February-March 2019 and imported into the ArcGIS software. To calculate areas of green/blue spaces for the cohort participants, the global coordinates within GIS data (WGS-84) need to be projected to two-dimensional space. The projection systems are different across countries due to variation in latitude and longitude. For the UK data, the British National Grid was used as this is most common projection method in the country. The Lambert Conformal Conic projection was used for the Mexico data as this system is adapted in the government postal code data and commonly used in North America. The Universal Transverse Mercator (UTM) system was used for the China (Beijing, 50N) and Dominican Republic data (Santo Domingo, 19N).

The coordinate information for participants’ residences was mapped onto the GIS data. Since the coordinates of the 10/66 China participants was coded in the GCJ-02 system, a R package (*GeoChina*) was used to transfer the GCJ-02 to WGS-84 coordinates so the coordinate data could appear in correct locations. Buffers with a radius of 400 or 800 metres were generated for all participants using the ArcGIS software (version 10.6.1). Based on the land use layer from Open Street Map, three types of green/blue spaces were identified: recreational green space (park, allotment and recreational space), nature (forest and natural reserve) and blue space (river, lake or sea). Percentages of these green and blue spaces within the 400m and 800m buffers were generated for all cohort participants.

**Local amenities in historical images**

Since Google Street View is more complete in the UK, a small number of random sample (N=108) were selected from the CFAS II cohort to investigate whether the identified amenities in 2019 could be found in earlier time points. The names and addresses of local amenities were entered to Google Maps and linked to historical Street View images back to 2008. The areas of parks were also reviewed using Google Earth, which provides historical satellite images. Since the CFAS II follow-up wave were carried out in 2011-2014, the review focused on whether the amenities could be found in images before 2014.

Table S1 reports the results by different types of local amenities. Just over 80% of amenities could be identified in the historical images corresponding to the time point of the CFAS II follow-up wave. A lower percentage (<70%) was found in café and GP surgery. The percentage was 81.5% in Cambridgeshire, 76.9% in Nottingham and 85.7% in Newcastle-upon-Type.

**Table S1.** Results of historical image review

|  | Café | Library | Movie theatre | Park | Post office | Convenience store | GP surgery | Pharmacy | Total |
| --- | --- | --- | --- | --- | --- | --- | --- | --- | --- |
| Can be found in historical images before 2014 | 12  (66.7) | 10  (90.9) | 5  (71.4) | 15  (93.8) | 12  (92.3) | 12  (92.3) | 11  (68.8) | 11  (78.6) | 88  (81.5) |
| Cannot be found in historical images before 2014 | 6  (33.3) | 1  (10.1) | 2  (28.6) | 1  (6.2) | 1  (7.7) | 1  (7.7) | 5  (31.2) | 3  (21.4) | 20  (18.5) |
| Total | 18 | 11 | 7 | 16 | 13 | 13 | 16 | 14 |  |

**Table S2.** Descriptive information on local amenities and green/blue spaces

|  | CFAS II | | 10/66 study | |
| --- | --- | --- | --- | --- |
|  | Median (IQR) | [min, max] | Median (IQR) | [min, max] |
| **Local amenities** |  |  |  |  |
| *Lifestyle* |  |  |  |  |
| Café |  |  |  |  |
| Distance: the nearest (km) | 0.49 (0.57) | [0.00, 7.24] | 0.17 (0.17) | [0.00, 4.70] |
| Number: 400m | 0 (1) | [0, 20] | 5 (5) | [0, 20] |
| Number: 800m | 2 (5) | [0, 20] | 20 (6) | [0, 20] |
| Library |  |  |  |  |
| Distance: the nearest (km) | 0.83 (0.77) | [0.00, 7.23] | 0.66 (0.73) | [0.07, 50.00] |
| Number: 400m | 0 (0) | [0, 5] | 0 (0) | [0, 2] |
| Number: 800m | 0 (1) | [0, 18] | 1 (2) | [0, 5] |
| Movie theatre |  |  |  |  |
| Distance: the nearest (km) | 3.54 (3.54) | [0.06, 50.00] | 0.58 (0.46) | [0.02, 6.00] |
| Number: 400m | 0 (0) | [0, 3] | 0 (1) | [0, 4] |
| Number: 800m | 0 (0) | [0, 4] | 1 (3) | [0, 8] |
| Park |  |  |  |  |
| Distance: the nearest (km) | 0.55 (0.62) | [0.00, 3.83] | 0.36 (0.44) | [0.00, 3.41] |
| Number: 400m | 0 (1) | [0, 5] | 1 (2) | [0, 16] |
| Number: 800m | 1 (3) | [0, 10] | 4 (8) | [0, 20] |
| *Daily life* |  |  |  |  |
| Post office |  |  |  |  |
| Distance: the nearest (km) | 0.60 (0.54) | [0.01, 50.00] | 0.82 (1.35) | [0.01, 50.00] |
| Number: 400m | 0 (1) | [0, 5] | 0 (0) | [0, 6] |
| Number: 800m | 1 (1) | [0, 12] | 0 (2) | [0, 11] |
| Convenience store |  |  |  |  |
| Distance: the nearest (km) | 0.43 (0.40) | [0.00, 50.00] | 0.18 (0.41) | [0.00, 4.45] |
| Number: 400m | 0 (1) | [0, 9] | 3 (16) | [0, 20] |
| Number: 800m | 2 (3) | [0, 20] | 8 (19) | [0, 20] |
| *Health services* |  |  |  |  |
| Doctor/hospital |  |  |  |  |
| Distance: the nearest (km) | 0.44 (0.41) | [0.00, 7.09] | 0.22 (0.32) | [0.00, 6.39] |
| Number: 400m | 0 (2) | [0, 16] | 2 (7) | [0, 19] |
| Number: 800m | 3 (5) | [0, 19] | 10 (15) | [0, 20] |
| Pharmacy |  |  |  |  |
| Distance: the nearest (km) | 0.51 (0.46) | [0.00, 50.00] | 0.17 (0.13) | [0.00, 4.81] |
| Number: 400m | 0 (1) | [0, 8] | 5 (5) | [0, 17] |
| Number: 800m | 1 (2) | [0, 13] | 19 (10) | [0, 20] |
| **Green/blue spaces** |  |  |  |  |
| Recreational green |  |  |  |  |
| Percentage: 400m (%) | 3.11 (7.31) | [0.00, 59.78] | 0.72 (2.28) | [0.00, 33.19] |
| Percentage: 800m (%) | 4.36 (6.34) | [0.00, 60.99] | 1.52 (2.06) | [0.00, 30.38] |
| Nature |  |  |  |  |
| Percentage: 400m (%) | 0.49 (2.79) | [0.00, 48.49] | 0.00 (0.00) | [0.00, 59.37] |
| Percentage: 800m (%) | 1.68 (3.45) | [0.00, 26.63] | 0.00 (0.26) | [0.00, 59.34] |
| Blue space |  |  |  |  |
| Percentage: 400m (%) | 0.00 (0.00) | [0.00, 29.00] | 0.00 (0.00) | [0.00, 14.75] |
| Percentage: 800m (%) | 0.00 (0.19) | [0.00, 18.48] | 0.00 (0.02) | [0.00, 22.60] |

**Table S3.** Tertile ranges for distance to the nearest local amenities and the percentage of green/blue spaces within 400m and 800m

|  | CFAS II | | 10/66 study | |
| --- | --- | --- | --- | --- |
| **Local amenities** | Tertile | Range (km) | Tertile | Range (km) |
| *Lifestyle* |  |  |  |  |
| Café | T1 | 0.00−0.36 | T1 | 0.00−0.12 |
|  | T2 | 0.36−0.71 | T2 | 0.13−0.23 |
|  | T3 | >0.71 | T3 | >0.23 |
| Library | T1 | 0.00−0.62 | T1 | 0.07−0.49 |
|  | T2 | 0.62−1.10 | T2 | 0.49−1.00 |
|  | T3 | >1.10 | T3 | >1.00 |
| Movie theatre | T1 | 0.06−2.50 | T1 | 0.02−0.44 |
|  | T2 | 2.50−4.66 | T2 | 0.44−0.73 |
|  | T3 | >4.66 | T3 | >0.73 |
| Park | T1 | 0.00−0.41 | T1 | 0.00−0.27 |
|  | T2 | 0.41−0.74 | T2 | 0.27−0.51 |
|  | T3 | >0.74 | T3 | >0.51 |
| *Daily life* |  |  |  |  |
| Post office | T1 | 0.01−0.43 | T1 | 0.01−0.59 |
|  | T2 | 0.43−0.78 | T2 | 0.59−1.06 |
|  | T3 | >0.78 | T3 | >1.06 |
| Convenience store | T1 | 0.00−0.31 | T1 | 0.00−0.10 |
|  | T2 | 0.31−0.56 | T2 | 0.10−0.35 |
|  | T3 | >0.56 | T3 | >0.36 |
| *Health services* |  |  |  |  |
| Doctor/hospital | T1 | 0.00−0.33 | T1 | 0.00−0.16 |
|  | T2 | 0.33−0.57 | T2 | 0.16−0.34 |
|  | T3 | >0.58 | T3 | >0.34 |
| Pharmacy | T1 | 0.00−0.38 | T1 | 0.00−0.13 |
|  | T2 | 0.38−0.68 | T2 | 0.14−0.21 |
|  | T3 | >0.68 | T3 | >0.21 |
|  |  |  |  |  |
| **Green/blue spaces** | Tertile | Range (%) | Tertile | Range (%) |
| *400m* |  |  |  |  |
| Recreational green | Low | 0.0−1.0 | Low | 0.0 |
|  | Middle | 1.1−6.0 | Middle | >0.0−1.9 |
|  | High | >6.0 | High | >1.9 |
| Nature | Low | 0.0 | Low | 0.0 |
|  | Middle | >0.0−1.7 | Middle | >0.0 |
|  | High | >1.7 | High | − |
| Blue space | None | 0.0 | None | 0.0 |
|  | Any | >0.0 | Any | >0.0 |
| *800m* |  |  |  |  |
| Recreational green | Low | 0.0−2.6 | Low | 0.0−0.7 |
|  | Middle | >2.6−6.6 | Middle | >0.7−2.1 |
|  | High | >6.6 | High | >2.1 |
| Nature | Low | 0.0−1.0 | Low | 0.0 |
|  | Middle | >1.0−3.2 | Middle | >0.0 |
|  | High | >3.2 | High | − |
| Blue space | None | 0.0 | None | 0.0 |
|  | Any | >0.0 | Any | >0.0 |

**S2. Additional results**

**Figure S2.** Maps of study areas

(A) CFAS II, UK

Cambridgeshire (rural; green), Nottingham (urban; blue), Newcastle-up-Type (urban; purple)

(B) The 10/66 study (China, Dominican Republic, Mexico)

China: Beijing


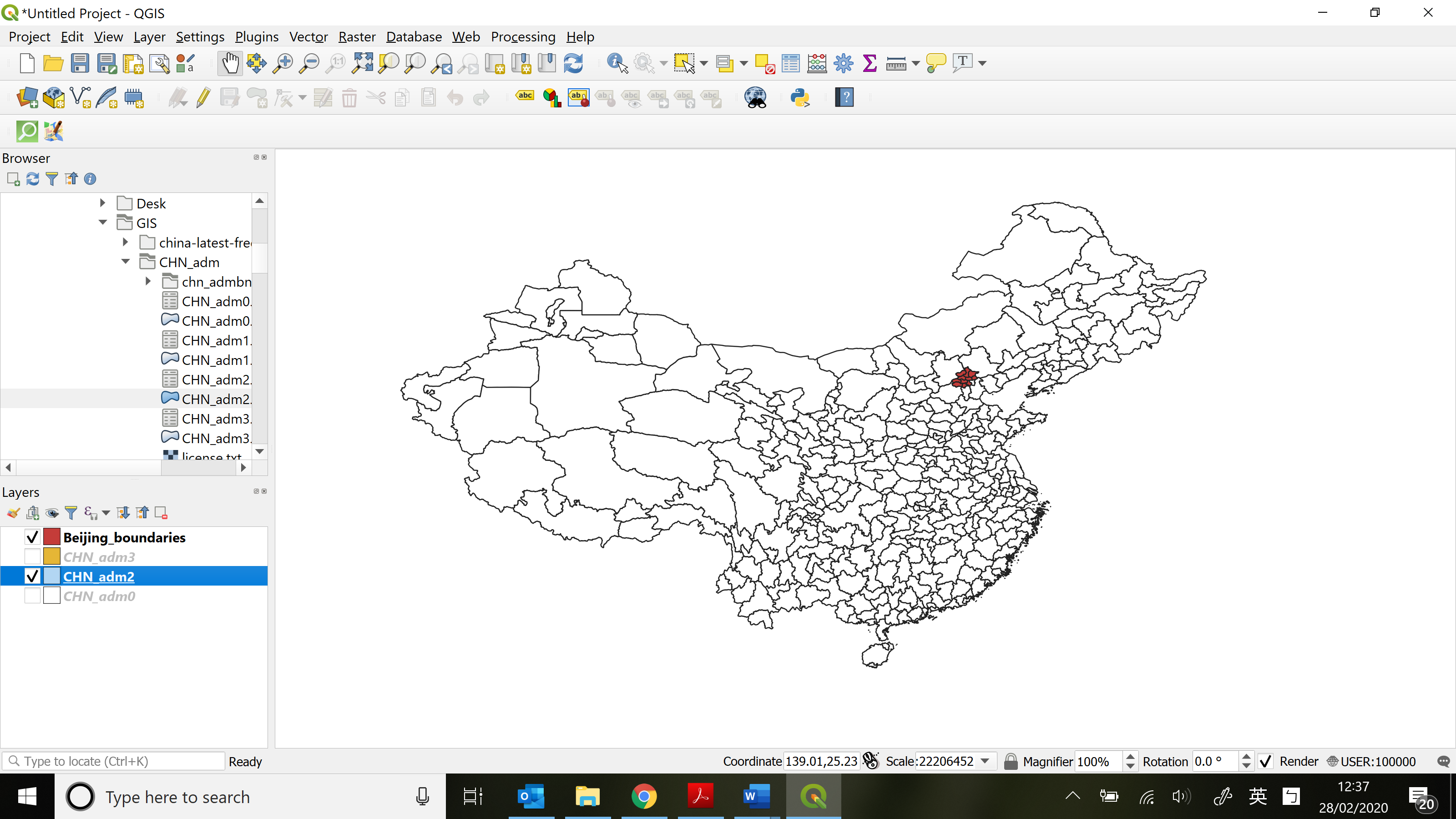


Dominican Republic: Santo Domingo


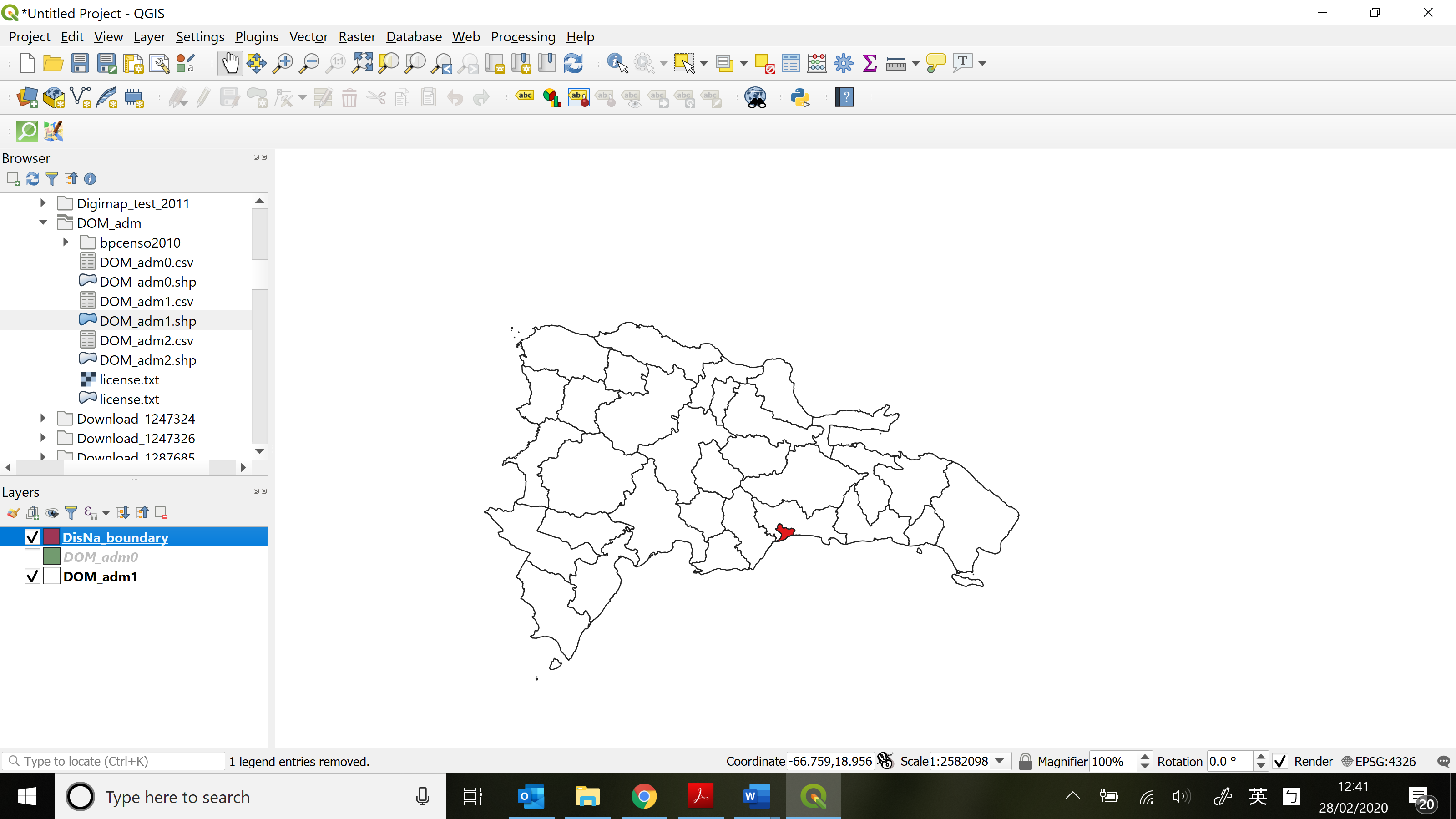


Mexico: Mexico City (urban, red) & Morelos (rural, orange)


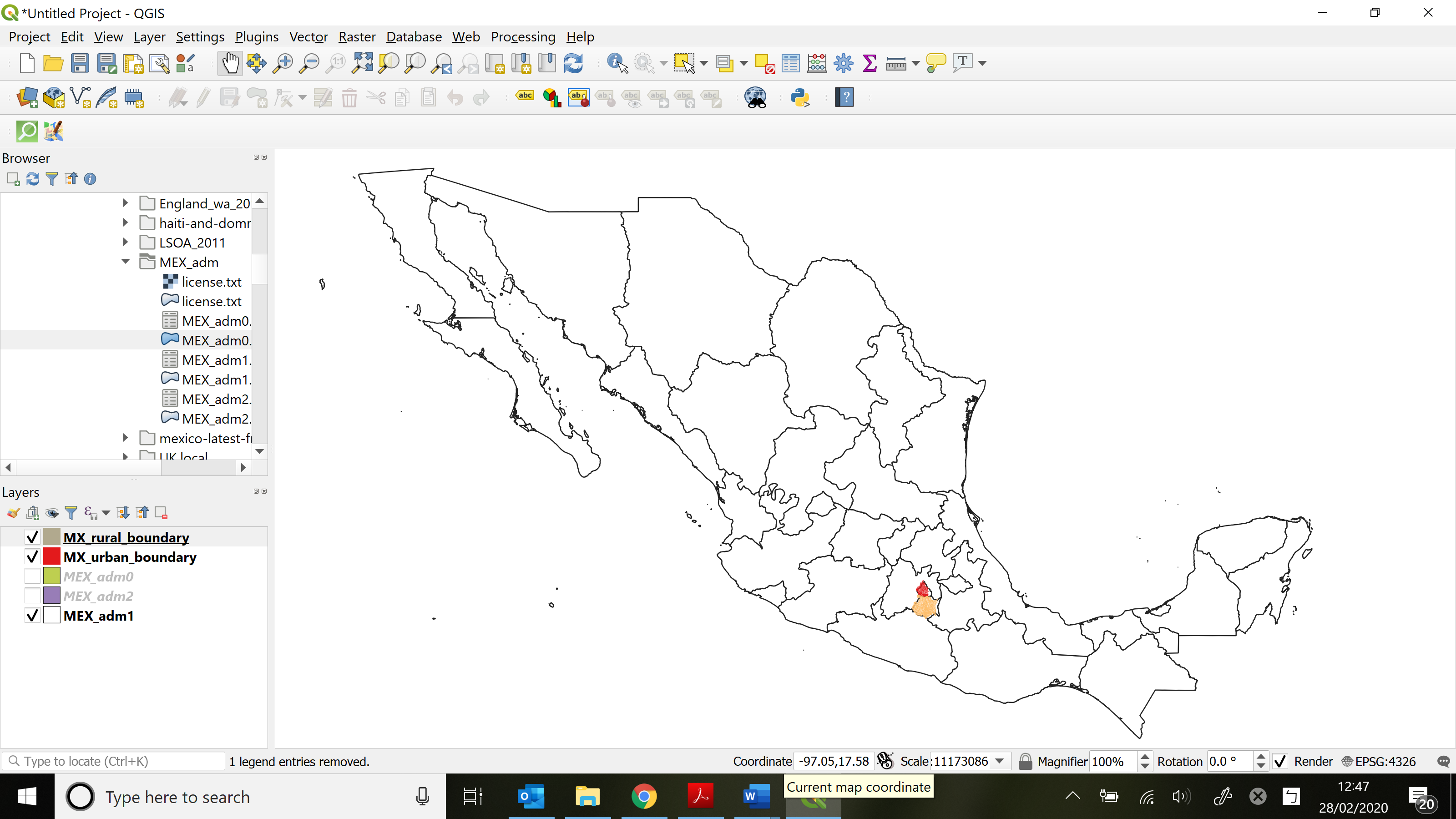

**Table S4.** The associations between sociodemographic factors, self-rated health and dementia (based on the GMS-AGECAT algorithm or the 10/66 algorithm) in CFAS II and the 10/66 study

|  | CFAS II | GMS-AGECAT | 10/66 | GMS-AGECAT | The 10/66 algorithm |
| --- | --- | --- | --- | --- | --- |
|  |  | OR (95% CI) |  | OR (95% CI) | OR (95% CI) |
| Age | Year | 1.12 (1.10, 1.14) | Year | 1.09 (1.08, 1.11) | 1.14 (1.12, 1.16) |
| Gender | Women | Ref. | Women | Ref. | Ref. |
|  | Men | 0.94 (0.70, 1.25) | Men | 0.54 (0.45, 0.66) | 0.80 (0.65, 0.98) |
| Education | High: 12+ years | Ref. | High: college or above | Ref. | Ref. |
|  | Middle: 10-11 years | 1.35 (0.87, 2.13) | Middle: secondary | 1.16 (0.58, 2.35) | 1.28 (0.72, 2.28) |
|  | Low: 9 or less years | 3.74 (2.39, 5.85) | Low: primary or none | 5.07 (2.81, 9.14) | 2.08 (1.27, 3.41) |
| Social class | I/II | Ref. |  |  |  |
|  | III-NM/III-M | 1.16 (0.79, 1.70) |  |  |  |
|  | IV/V | 2.58 (1.70, 3.92) |  |  |  |
| Number of |  |  | 6-7 | Ref. | Ref. |
| household assets |  |  | 4-5 | 1.39 (1.15, 1.68) | 1.53 (1.24, 1.88) |
|  |  |  | 0-3 | 3.06 (2.43, 3.86) | 2.10 (1.61, 2.73) |
| Self-rated health | Excellent | Ref. | Very good | Ref. | Ref. |
|  | Good | 1.20 (0.77, 1.85) | Good | 1.27 (0.97, 1.67) | 1.23 (0.89, 1.69) |
|  | Fair | 1.84 (1.14, 2.95) | Moderate | 0.99 (0.77, 1.29) | 1.23 (0.91, 1.66) |
|  | Poor | 3.35 (1.82, 6.17) | Bad | 1.46 (0.97, 2.21) | 2.00 (1.28, 3.13) |
|  |  |  | Very bad | 0.62 (0.26, 1.83) | 1.67 (0.70, 3.98) |

Dementia diagnosis in CFAS II was based on the GMS-AGECAT algorithm and the 10/66 study identified dementia cases using the GMS-AGECAT algorithm and the 10/66 dementia

**Table S5.** The associations between local amenities, green and blue spaces within 400m or 800m buffers and dementia based on the GMS-AGECAT algorithm and the 10/66 dementia diagnostic algorithm (adjusted for age, gender, education, social class/number of assets, self-rated health and centre/site)

|  |  | CFAS II  (GMS-AGECAT) | |  | 10/66  (GMS-AGECAT) | (the 10/66 algorithm) |
| --- | --- | --- | --- | --- | --- | --- |
| **Local amenities** |  | N (%) | OR (95% CI) | N (%) | OR (95% CI) | OR (95% CI) |
| *400m* |  |  |  |  |  |  |
| *Lifestyle* | Any | 2864 (57.8) | - | 2998 (88.5) | - | - |
|  | None | 2091 (42.2) | 0.92 (0.65, 1.30) | 388 (11.5) | 1.16 (0.83, 1.60) | 1.68 (1.12, 2.52) |
| *Daily life* | Any | 2793 (56.4) | - | 2386 (70.5) | - | - |
|  | None | 2162 (23.6) | 1.13 (0.81, 1.60) | 1000 (29.5) | 1.20 (0.96, 1.50) | 1.18 (0.91, 1.52) |
| *Healthcare* | Any | 2589 (52.3) | - | 3027 (89.4) | - | - |
|  | None | 2366 (47.7) | 0.96 (0.68, 1.36) | 359 (10.6) | 0.96 (0.68, 1.34) | 1.30 (0.87, 1.95) |
| *All amenities* | Any | 3930 (79.3) | - | 3161 (93.4) | - | - |
|  | None | 1025 (20.7) | 0.96 (0.61, 1.51) | 225 0(6.6) | 1.16 (0.80, 1.68) | 1.57 (1.01, 2.42) |
| *800m* |  |  |  |  |  |  |
| *Lifestyle* | Any | 4405 (88.9) | - | 3137 (92.7) | - | - |
|  | None | 550 (11.1) | 0.78 (0.42, 1.48) | 249 0(7.4) | 1.19 (0.84, 1.69) | 1.75 (1.16, 2.66) |
| *Daily life* | Any | 4446 (89.7) | - | 3106 (91.7) | - | - |
|  | None | 509 (10.3) | 1.16 (0.63, 2.14) | 280 0(8.3) | 1.27 (0.92, 1.75) | 1.44 (0.99, 2.09) |
| *Healthcare* | Any | 4234 (85.5) | - | 3189 (94.2) | - | - |
|  | None | 721 (14.5) | 1.21 (0.72, 2.02) | 197 0(5.8) | 1.12 (0.77, 1.65) | 1.53 (0.98, 2.39) |
| *All amenities* | Any | 4804 (97.0) | - | 3251 (96.0) | - | - |
|  | None | 151 0(3.0) | 1.09 (0.35, 3.09) | 135 0(4.0) | 0.92 (0.60, 1.41) | 1.47 (0.91, 2.40) |
| **Green/blue spaces** |  |  |  |  |  |  |
| *400m* | Any | 4632 (93.5) | - | 2367 (69.9) | - | - |
|  | None | 323 0(6.5) | 1.49 (0.81, 2.75) | 1019 (30.1) | 1.01 (0.80, 1.28) | 1.10 (0.85, 1.44) |
| *800m* | Any | 4949 (99.9) | - | 3113 (91.9) | - | - |
|  | None | 6 0(0.1) | NA | 273 0(8.1) | 0.80 (0.55, 1.15) | 0.87 (0.59, 1.28) |

**Table S6.** The associations between local amenities, green and blue spaces and dementia (based on the GMS-AGECAT algorithm) across different study sites (adjusted for age, gender, education and social class/assets)

| **Local amenities** |  | CFAS II |  |  | 10/66 |  |  |  |
| --- | --- | --- | --- | --- | --- | --- | --- | --- |
|  |  | Cambridgeshire | Newcastle | Nottingham | China | DR | Mexico-urban | Mexico-rural |
| *Lifestyle* |  | OR (95% CI) | OR (95% CI) | OR (95% CI) | OR (95% CI) | OR (95% CI) | OR (95% CI) | OR (95% CI) |
| Café | T1 | - | - | - | - | - | - | - |
|  | T2 | 1.38 (0.62, 3.07) | 1.00 (0.51, 1.96) | 0.76 (0.40, 1.46) | 1.52 (0.78, 2.94) | 0.81 (0.58, 1.12) | 1.23 (0.70, 2.17) | 3.22 (1.12, 9.29) |
|  | T3 | 0.81 (0.37, 1.78) | 2.03 (1.05, 3.94) | 0.87 (0.42, 1.79) | 1.18 (0.59, 2.34) | 0.91 (0.36, 2.30) | 2.37 (1.42, 3.95) | 3.10 (1.15, 8.36) |
| Library | T1 | - | - | - | - | - | - | - |
|  | T2 | 0.94 (0.43, 2.04) | 1.63 (0.85, 3.13) | 0.56 (0.28, 1.11) | 0.92 (0.43, 1.98) | 1.02 (0.54, 1.87) | 2.77 (1.56, 4.90) | 1.10 (0.49, 2.46) |
|  | T3 | 0.82 (0.42, 1.61) | 1.70 (0.83, 3.46) | 1.03 (0.51, 2.06) | 1.00 (0.54, 1.87) |  | 2.31 (1.24, 4.28) | 1.13 (0.60, 2.11) |
| Movie theatre | T1 | - | - | - | - | - | - | - |
|  | T2 | 2.01 (0.62, 6.56) | 1.11 (0.58, 2.15) | 0.81 (0.44, 1.47) | 1.24 (0.72, 2.14) | 1.23 (0.85, 1.78) | 0.78 (0.51, 1.18) | 0.53 (0.17, 1.70) |
|  | T3 | 1.67 (0.81, 3.46) | 1.12 (0.54, 2.35) | 0.96 (0.36, 2.54) | 1.34 (0.57, 3.14) | 1.17 (0.77, 1.77) | 0.62 (0.36, 1.06) | 1.11 (0.60, 2.06) |
| Park | T1 | - | - | - | - | - | - | - |
|  | T2 | 0.55 (0.25, 1.23) | 0.74 (0.38, 1.43) | 1.30 (0.71, 2.37) | 0.83 (0.31, 2.24) | 0.84 (0.61, 1.16) | 0.78 (0.51, 1.18) | 1.06 (0.55, 2.04) |
|  | T3 | 0.45 (0.22, 0.90) | 0.61 (0.30, 1.21) | 0.83 (0.29, 2.36) | 1.07 (0.44, 2.63) | 0.58 (0.14, 2.38) | 0.53 (0.30, 0.91) | 1.21 (0.71, 2.08) |
| *Daily life* |  |  |  |  |  |  |  |  |
| Post office | T1 | - | - | - | - | - | - | - |
|  | T2 | 1.20 (0.53, 2.70) | 1.43 (0.72, 2.84) | 1.46 (0.74, 2.90) | 0.76 (0.36, 1.62) | 1.24 (0.78, 1.98) | 0.95 (0.22, 3.97) | 0.90 (0.46, 1.78) |
|  | T3 | 1.73 (0.87, 3.45) | 1.47 (0.73, 2.98) | 1.33 (0.62, 2.88) |  | 1.45 (0.76, 2.78) | 2.81 (1.42, 5.59) | 1.05 (0.70, 1.58) |
| Convenience | T1 | - | - | - | - | - | - | - |
| store | T2 | 1.03 (0.46, 2.30) | 0.85 (0.44, 1.62) | 1.25 (0.65, 2.40) | 0.89 (0.48, 1.66) | 1.19 (0.57, 2.47) | 0.78 (0.53, 1.15) | 1.31 (0.75, 2.28) |
|  | T3 | 1.16 (0.56, 2.40) | 0.98 (0.50, 1.93) | 1.29 (0.61, 2.75) |  | 1.24 (0.61, 2.51) | 0.72 (0.22, 2.35) | 1.49 (0.87, 2.55) |
| *Healthcare* |  |  |  |  |  |  |  |  |
| Doctor | T1 | - | - | - | - | - | - | - |
|  | T2 | 1.11 (0.51, 2.42) | 0.93 (0.50, 1.73) | 0.86 (0.44, 1.69) | 0.99 (0.58, 1.68) | 0.94 (0.65, 1.38) | 0.94 (0.63, 1.41) | 0.05 (0.00, 1.00) |
|  | T3 | 1.00 (0.48, 2.07) | 0.85 (0.41, 1.77) | 0.96 (0.47, 1.94) |  | 1.09 (0.73, 1.63) | 1.12 (0.58, 2.15) | 0.08 (0.01, 1.12) |
| Pharmacy | T1 | - | - | - | - | - | - | - |
|  | T2 | 1.59 (0.71, 3.60) | 0.78 (0.44, 1.40) | 1.24 (0.64, 2.39) | 1.30 (0.75, 2.27) | 1.10 (0.76, 1.59) | 0.68 (0.43, 1.05) | 0.94 (0.48, 1.83) |
|  | T3 | 1.08 (0.51, 2.31) | 0.45 (0.17, 1.17) | 0.87 (0.42, 1.82) | 0.89 (0.40, 1.95) | 1.10 (0.75, 1.63) | 0.87 (0.54, 1.42) | 0.88 (0.51, 1.52) |
| **Green/blue spaces** | | | | | | | | |
| *400m* |  |  |  |  |  |  |  |  |
| Recreational | L | - | - | - | - | - | - | - |
| green | M | 1.59 (0.85, 2.98) | 0.79 (0.37, 1.67) | 0.65 (0.28, 1.53) | 1.67 (0.79, 3.49) | 1.16 (0.52, 2.60) | 1.74 (1.15, 2.63) | 0.81 (0.52, 1.28) |
|  | H | 0.97 (0.41, 2.34) | 1.28 (0.70, 2.40) | 1.22 (0.58, 2.54) | 0.49 (0.22, 1.08) | 1.50 (0.69, 3.27) | 0.72 (0.39, 1.33) |  |
| Nature | L | - | - | - | - | - | - | - |
|  | M | 0.61 (0.30, 1.23) | 1.21 (0.60, 2.44) | 1.67 (0.83, 3.36) |  |  | 0.56 (0.33, 0.94) | 1.26 (0.89, 1.78) |
|  | H | 1.19 (0.57, 2.47) | 0.73 (0.38, 1.41) | 1.39 (0.70, 2.75) |  |  |  |  |
| Blue space | None | - | - | - | - | - | - | - |
|  | Any | 0.88 (0.43, 1.80) | 1.80 (0.83, 3.90) | 0.89 (0.43, 1.88) |  | 1.11 (0.73, 1.69) | 0.71 (0.13, 3.76) |  |
| *800m* |  |  |  |  |  |  |  |  |
| Recreational | L | - | - | - | - | - | - | - |
| green | M | 1.31 (0.71, 2.42) | 1.28 (0.58, 2.84) | 1.64 (0.52, 5.13) | 0.90 (0.43, 1.85) | 1.09 (0.47, 2.49) | 1.46 (0.90, 2.37) | 0.48 (0.08, 2.68) |
|  | H | 0.52 (0.06, 4.55) | 1.38 (0.65, 2.94) | 1.67 (0.55, 5.03) | 0.98 (0.55, 1.74) | 1.03 (0.44, 2.37) | 0.62 (0.33, 1.17) |  |
| Nature | L | - | - | - | - | - | - | - |
|  | M | 1.18 (0.63, 2.20) | 0.76 (0.38, 1.52) | 0.88 (0.42, 1.85) |  |  | 0.83 (0.56, 1.22) | 1.06 (0.65, 1.74) |
|  | H | 0.47 (0.18, 1.24) | 0.64 (0.33, 1.26) | 1.39 (0.72, 2.67) |  |  |  |  |
| Blue space | None | - | - | - | - | - | - | - |
|  | Any | 0.93 (0.51, 1.71) | 0.94 (0.53, 1.65) | 1.02 (0.58, 1.80) |  | 1.19 (0.85, 1.65) | 1.02 (0.56, 1.87) |  |

T1, T2, T3: first, second and third tertile of distance to the nearest local amenities; H, M, L: high, middle and low percentages of green and blue spaces by tertiles

**Table S7.** The stratified associations between local amenities and dementia (based on the GMS-AGECAT algorithm) by socioeconomic levels (adjusted for age, gender, education, self-rated health and centre/site)

|  |  | CFAS II: social class |  |  |
| --- | --- | --- | --- | --- |
|  |  | High (I/II) | Middle (III-NM/III-M) | Low (IV/V) |
| Local amenities |  | OR (95% CI) | OR (95% CI) | OR (95% CI) |
| Post office | T1 | - | - | - |
|  | T2 | 1.55 (0.62, 3.87) | 1.60 (0.89, 2.88) | 0.85 (0.39, 1.82) |
|  | T3 | 1.88 (0.78, 4.54) | 1.49 (0.80, 2.78) | 1.26 (0.60, 2.65) |
| Park | T1 | - | - |  |
|  | T2 | 1.12 (0.48, 2.62) | 1.11 (0.65, 1.91) | 0.51 (0.24, 1.08) |
|  | T3 | 1.02 (0.43, 2.45) | 0.57 (0.29, 1.12) | 0.50 (0.23, 1.07) |
|  |  |  |  |  |
|  |  | 10/66: Number of assets |  |  |
|  |  | High (6-7 assets) | Middle (4-5 assets) | Low (0-3 assets) |
|  |  | OR (95% CI) | OR (95% CI) | OR (95% CI) |
| Post office | T1 | - | - | - |
|  | T2 | 1.61 (1.08, 2.38) | 0.69 (0.44, 1.08) | 0.83 (0.37, 1.86) |
|  | T3 | 2.14 (1.42, 3.24) | 0.78 (0.49, 1.23) | 0.94 (0.39, 2.25) |
| Café | T1 | - | - | - |
|  | T2 | 1.00 (0.72, 1.38) | 0.91 (0.60, 1.38) | 2.16 (1.07, 4.35) |
|  | T3 | 1.20 (0.83, 1.75) | 1.32 (0.84, 2.10) | 2.75 (1.22, 6.17) |

T1, T2, T3: first, second and third tertile of distance to the nearest local amenities

**Table S8.** Results of logistic regression: the associations between local amenities and dementia (based on the GMS-AGECAT algorithm and the 10/66 dementia diagnostic algorithm) adjusted for age, gender, education, self-rated health and centre/site

|  |  | Complete case | Multiple imputation |
| --- | --- | --- | --- |
|  |  | OR (95% CI) | OR (95% CI) |
| CFAS II (GMS-AGECAT) |  |  |  |
| Park | T1 | - | - |
|  | T2 | 0.90 (0.62, 1.30) | 0.90 (0.63, 1.28) |
|  | T3 | 0.64 (0.41, 1.00) | 0.68 (0.45, 1.03) |
| Post office | T1 | - | - |
|  | T2 | 1.27 (0.85, 1.89) | 1.28 (0.88, 1.87) |
|  | T3 | 1.47 (0.98, 2.22) | 1.47 (1.00, 2.17) |
|  |  |  |  |
| 10/66 (GMS-AGECAT) |  |  |  |
| Café | T1 | - | - |
|  | T2 | 1.06 (0.85, 1.33) | 1.06 (0.85, 1.33) |
|  | T3 | 1.38 (1.05, 1.81) | 1.38 (1.05, 1.82) |
| Post office | T1 | - | - |
|  | T2 | 1.10 (0.84, 1.45) | 1.11 (0.85, 1.46) |
|  | T3 | 1.37 (1.02, 1.84) | 1.38 (1.03, 1.86) |
|  |  |  |  |
| 10/66 (10/66 algorithm) |  |  |  |
| Café | T1 | - | - |
|  | T2 | 0.87 (0.67, 1.12) | 0.85 (0.66, 1.10) |
|  | T3 | 1.30 (0.95, 1.77) | 1.28 (0.94, 1.74) |
| Convenience store | T1 | - | - |
|  | T2 | 1.24 (0.92, 1.67) | 1.23 (0.91, 1.67) |
|  | T3 | 1.42 (1.02, 1.98) | 1.39 (0.99, 1.95) |
| Hospital | T1 | - | - |
|  | T2 | 1.06 (0.83, 1.36) | 1.05 (0.82, 1.36) |
|  | T3 | 1.20 (0.86, 1.68) | 1.19 (0.84, 1.68) |

T1, T2, T3: first, second and third tertile of distance to the nearest local amenities
